# Flaviviral NS4b, chameleon and jack‐in‐the‐box roles in viral replication and pathogenesis, and a molecular target for antiviral intervention

**DOI:** 10.1002/rmv.1835

**Published:** 2015-04-01

**Authors:** Joanna Zmurko, Johan Neyts, Kai Dallmeier

**Affiliations:** ^1^ KU Leuven, Rega Institute for Medical Research, Department of Microbiology and ImmunologyLaboratory of Virology and Chemotherapy

## Abstract

Dengue virus and other flaviviruses such as the yellow fever, West Nile, and Japanese encephalitis viruses are emerging vector‐borne human pathogens that affect annually more than 100 million individuals and that may cause debilitating and potentially fatal hemorrhagic and encephalitic diseases. Currently, there are no specific antiviral drugs for the treatment of flavivirus‐associated disease. A better understanding of the flavivirus–host interactions during the different events of the flaviviral life cycle may be essential when developing novel antiviral strategies. The flaviviral non‐structural protein 4b (NS4b) appears to play an important role in flaviviral replication by facilitating the formation of the viral replication complexes and in counteracting innate immune responses such as the following: (i) type I IFN signaling; (ii) RNA interference; (iii) formation of stress granules; and (iv) the unfolded protein response. Intriguingly, NS4b has recently been shown to constitute an excellent target for the selective inhibition of flavivirus replication. We here review the current knowledge on NS4b. © 2015 The Authors. *Reviews in Medical Virology* published by John Wiley & Sons Ltd.

**Abbreviations used**
AGO: Argonaute protein
C: capsid protein
CDC: Centers for Disease Control and Prevention
DENV: dengue virus
DF: dengue fever
dsRNA: double‐stranded RNA
DHF/DSS: dengue hemorrhagic fever/dengue shock syndrome
elF2α: eukaryotic elongation factor 2α
E: envelope protein
FRET: fluorescence resonance energy transfer
JAK‐STAT: Janus kinase‐signal transducer and activator of transcription
IRE‐1: inositol‐requiring protein 1
ISGs: IFN‐stimulated genes
IRF3/7: IFN regulatory factor 3/7
JEV: Japanese encephalitis virus
prM/M: membrane
C57BL/6 MEFs: mouse embryonic fibroblast
C3H/He MEFs: wild‐type murine embryonic fibroblasts
MTase: methyltransferase
NGC: New Guinea C
NITD: Novartis Institute for Tropical Diseases
PIAS‐1: protein inhibitor of activated STAT‐1
PTP‐1B: the protein tyrosine phosphatase 1B
PKR: protein kinase R
PGK1: phosphoglycerate kinase 1
RC: replication complexes
RdRp: RNA‐dependent RNA polymerase
RISC: RNA‐induced silencing complex
RNAi: RNA interference
RNase: endoribonuclease
shRNA: small hairpin RNA
STING: stimulator of IFN genes proteins
RIG‐I: retinoic acid‐inducible gene I
SUMO: small ubiquitin‐like modifier
TLR3/7: Toll‐like receptor 3/7
TBEV: tick‐borne encephalitis virus
Ube2i: ubiquitin‐conjugating enzyme E2I
UPR: unfolded protein response
WNV: West Nile virus
YFV: yellow fever virus
Xbp‐1: X‐box binding protein 1
WHO: World Health Organization

## Introduction

The genus *Flavivirus* comprises over 70 members, including important human pathogens such as dengue virus (DENV), yellow fever virus (YFV), West Nile virus (WNV), Japanese encephalitis virus (JEV), and tick‐borne encephalitis virus (TBEV). DENV is considered to be the most prevalent mosquito‐borne viral disease, endemic in over 100 countries with over three billion people at direct risk of infection 1. An estimated 390 million people become infected with DENV, of which 96 million become severely sick and half a million people develop dengue hemorrhagic fever/dengue shock syndrome leading to over 22 000 deaths annually 2. YFV is endemic in 44 countries in the tropical regions of Africa and South America 3 and causes acute febrile hemorrhagic yellow fever disease of humans and other primates 4. Despite the availability of a very efficient live‐attenuated (17D) vaccine 5, many people in endemic countries are not yet vaccinated, so hundred thousands of cases of yellow fever continue to occur, which result in 30 000 deaths each year 6. WNV is the most widespread arbovirus in the world that can cause severe neurological diseases including encephalitis and meningoencephalitis 7, 8. WNV introduction in 1999 to the USA demonstrates the ability of mosquito‐borne flaviviruses to cause global epidemics in previously non‐affected regions 9. Infection with JEV may cause a debilitating inflammation of the CNS. The disease is prevalent in much of Asia and the Western Pacific, with over four billion people at risk of infection in the region 10. Despite the availability of a number of safe vaccines 11, outbreaks of JEV occur regularly. Mortality is 25% with a specifically high mortality and disease burden in children in poorly developed countries of Southeast Asia 12.

In the last decennium, potent drugs have been developed for the treatment of infection with herpesviruses, human immunodeficiency virus, hepatitis B and C, and influenza. Highly potent and safe inhibitors of HCV replication (which belongs together with the flaviviruses to the family of the *Flaviviridae*) have been developed. The combined use of two or more drugs in one single tablet allows the achievement of >95% sustained virological response (cure) in patients chronically infected with the HCV 13. This provides encouragement to develop highly potent and safe flavivirus inhibitors that can be used for the treatment (and in some cases even prophylaxis) of (acute) flavivirus infection of non‐immunized populations during outbreaks in endemic regions, and these may be used by travelers 14, 15, 16.

## Expression, Post‐translational Processing, Structure, Localization, and Interaction of NS4b with Other Viral Proteins

**Figure 1:**
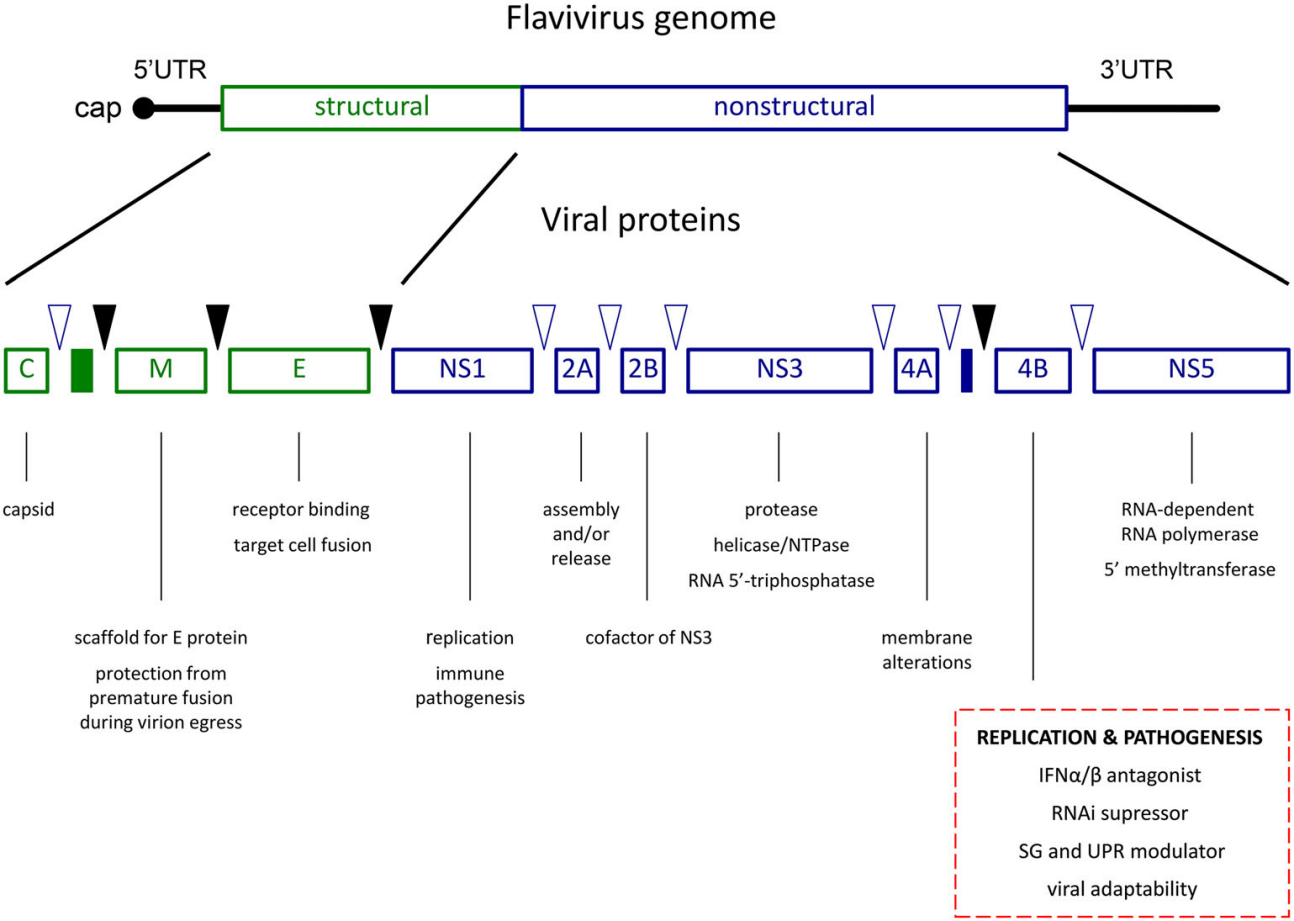
Genome organization and viral protein expression of flaviviruses. The flaviviral genome consists of a single‐stranded RNA of about 11 kb that encodes, in plus‐sense orientation, for a single ORF nested between highly structured 5′ and 3′ UTR. The ORF is translated at the rough ER as a single polyprotein, which is processed co‐translationally and post‐translationally by viral (open blue triangles) and host (black triangles) proteases into 10 mature viral proteins, of which three are structural proteins (C, core; M, membrane; and E, envelope) and seven are non‐structural (NS) proteins (NS1, NS2A, NS2B, NS3, NS4A, NS4B, and NS5). The nascent polypeptide chain is co‐translationally inserted into the ER membrane by the activity of several internal transfer/stop peptides such as the 2K peptide preceding the NS4B protein sequence (blue rectangle). The molecular function of many NS proteins is only poorly understood. Functions associated with NS4b has been implemented in viral replication and pathogenesis (modified from Sampath and Padmanabhan Antiviral Res. 2009 19)

The flavivirus genome is a plus‐sense, single‐stranded RNA of about 11 000 nucleotides, consisting of a 5′ untranslated region (UTR) carrying a canonical cap structure, a single ORF, and a 3′ UTR, which is (with the exception of TBEV) not polyadenylated but instead forms a functionally equivalent complex RNA fold 17. The single ORF encodes a single polyprotein that is co‐translationally and post‐translationally processed by viral and host proteases into 10 mature viral proteins—three structural proteins C, prM/M, and E and seven non‐structural (NS) proteins NS1, NS2a, NS2b, NS3, NS4a, NS4b, and NS5 18 (Figure 1). The NS proteins are involved in virus replication and the formation of the membranous replication complexes (RCs) 19. NS3 functions as a protease (with NS2b as a cofactor), a nucleotide triphosphatase, an RNA triphosphatase, and a helicase 20, 21, 22. NS5 encodes functional methyltransferase and an RNA‐dependent RNA polymerase (RdRp) 23, 24, 25, 26, 27. NS1 is physically associated with the RC; the secreted form is used as a marker for DENV infection. Moreover, specific interactions with a wide range of host cell components have been reported for both the excreted (sNS1) and membrane‐associated (mNS1) forms. These may be involved in the pathogenesis of the flavivirus disease (recent review on NS1 protein) 28. Similarly, the NS4b appears as a chameleon with a plethora of cellular and molecular functions assigned.

In DENV‐infected cells, NS4b is first cleaved to a polypeptide with an Mr of 30 000 (2K‐NS4b) by the activity of the viral NS2b–NS3 protease and is next post‐translationally modified by host cell signalase to the final product with an Mr of 27 000–28 000 (mature NS4b). The 2K peptide is essential for a proper co‐translational membrane insertion and protein folding 29, 30, 31, yet to some extent, it can be functionally exchanged by unrelated signal peptides, such as the murine MHC‐1 signal peptide KB 32.

**Figure 2:**
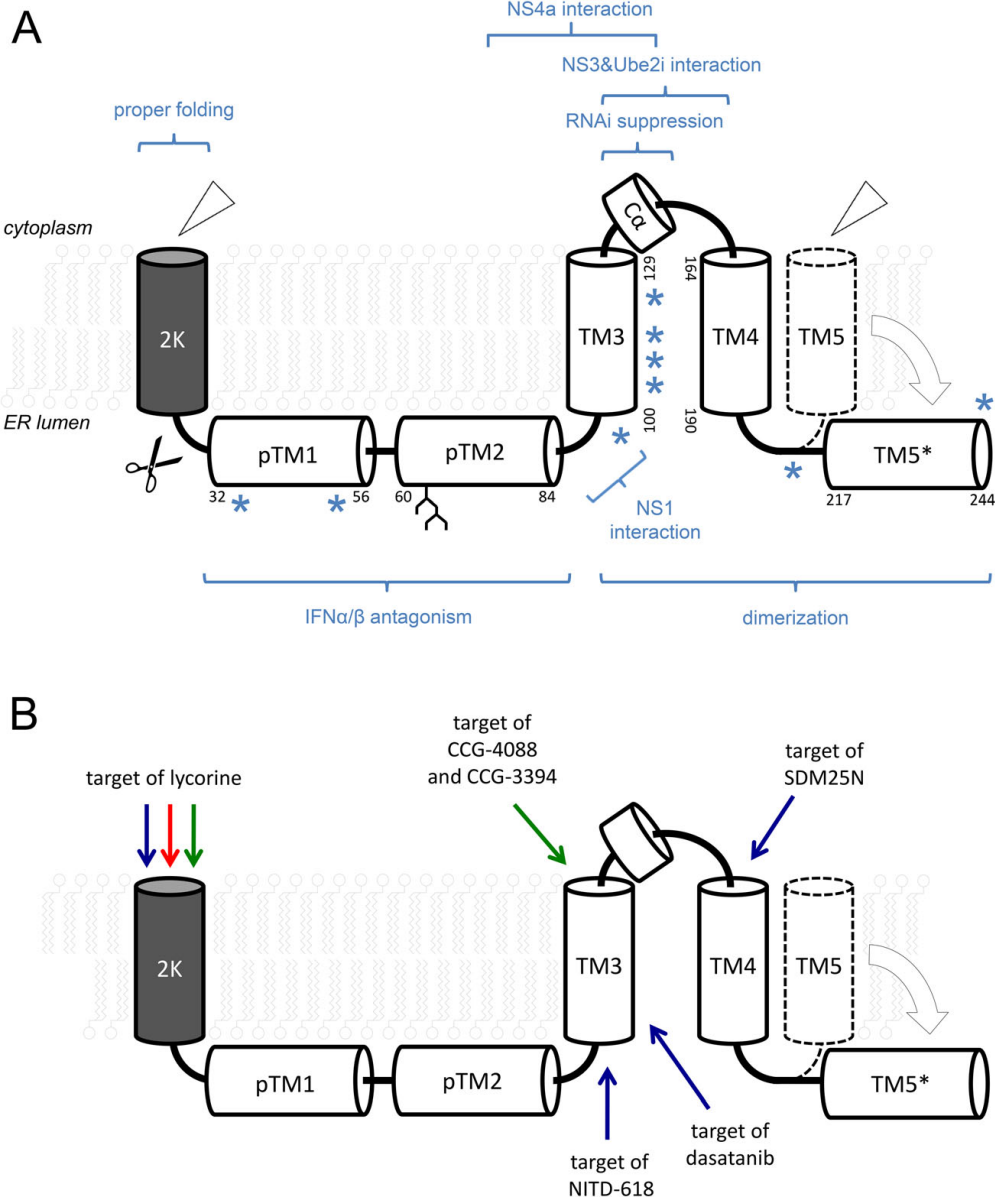
(A) Structural and functional topology of NS4b. The flaviviral NS4b protein is an integral membrane protein with several transmembrane transversions. Apart from the N‐terminal 2K signal peptide that is cleaved off after co‐translational insertion of the nascent NS4b polypeptide in the ER membrane by cellular signalase (*scissors*), the mature NS4b protein contains five major helical domains, three of which are experimentally proven transmembrane helices (TM3–5) and two predicted ones (pTM1 and pTM2), yet more likely residing in the ER lumen. The pTMD2 may be N‐glucosylated (*branched tree*). After proteolytic cleavage from NS5 protein by the viral NS2b/3 protease (*open triangle*), TM5 translocates to the ER luminal side (TM5*). Asterisks (*) indicate sites where mutations have been shown to largely influence viral virulence. The N‐terminal half of NS4b has been shown to interfere with IFN‐α/β signaling mediated by STAT‐1, the C‐terminal half implemented in NS4b dimerization and RNAi suppression. A short α‐helix in the cytoplasmic loop between TM3 and TM4 (Cα) interacts with NS3, and it might interact with Ube2i. Amino acids 84–146 were shown to interact with NS4a protein. Amino acid positions according to DENV‐2 NS4b (248 amino acids) (modified from Miller *et al*. 31 and Zou *et al*. 42). (B) Resistance to anti‐flavivirus inhibitors mapping to NS4b. Small molecular inhibitors of flavivirus replication (putatively) targeting NS4b select for characteristic resistance‐conferring mutations. Activity against DENV, blue arrows; against YFV, green arrows; against WNV, red arrow

**Figure 3:**
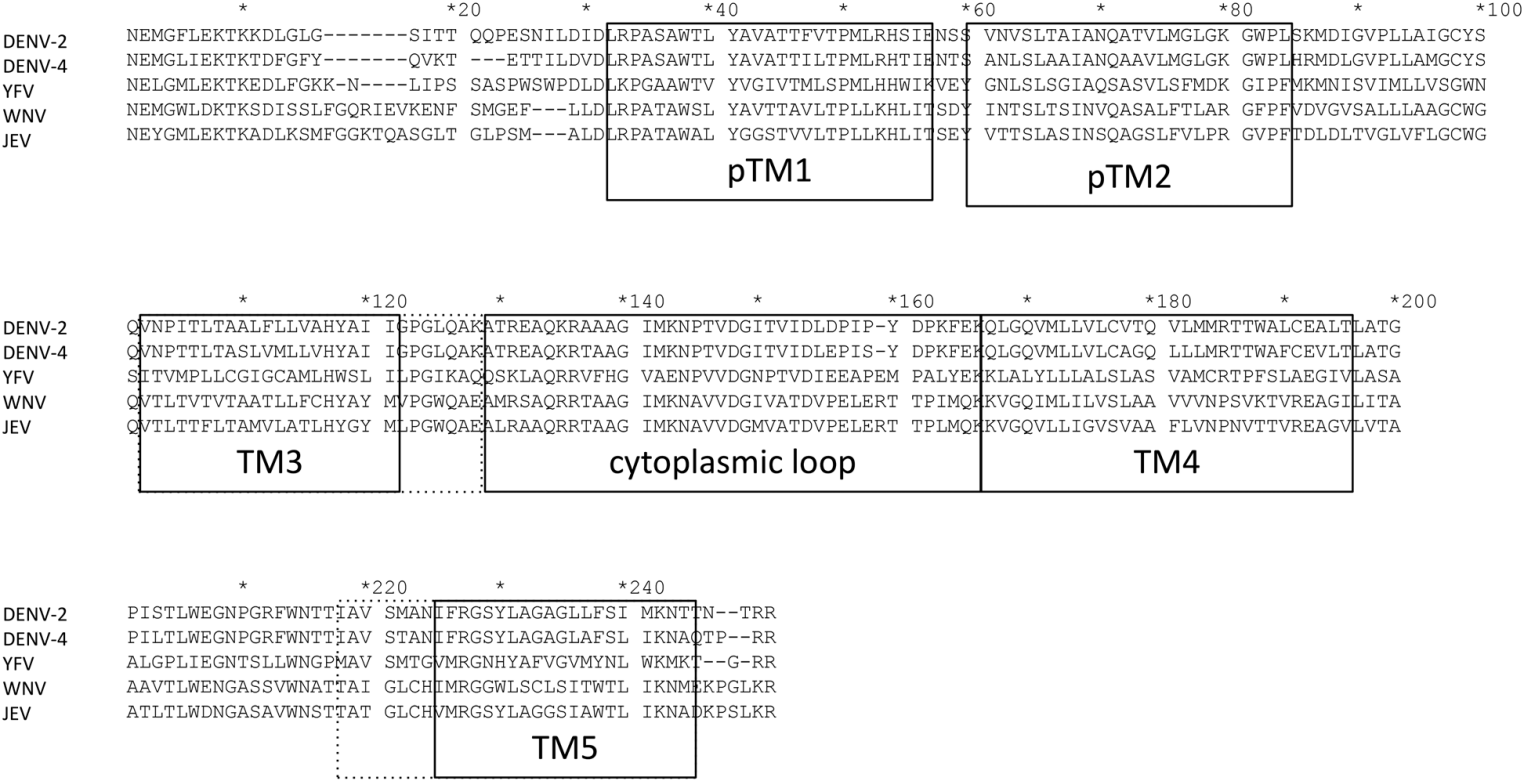
Alignment of flaviviral NS4b protein sequences. Representative members of the DENV‐2 (strain New Guinea C, GenBank AF038403), DENV‐4 (strain Dominica, GenBank AY648301), YFV (vaccine strain YFV‐17D, GenBank X03700), WNV (strain NY99, GenBank NC_009942), and JEV (vaccine strain SA14‐14‐2, GenBank D90195.1) were chosen to serve as reference for the NS4b mutations listed in Tables 1 and 2. A more comprehensive alignment including an exhaustive list of Flaviviruses can be found in Wicker *et al*. 60. The assignment of secondary structures as depicted in Figure 2A and B according to Miller *et al*. 30, Xie *et al*. 29, and Zou *et al*. 41 (*dotted lines*)

**Figure 4:**
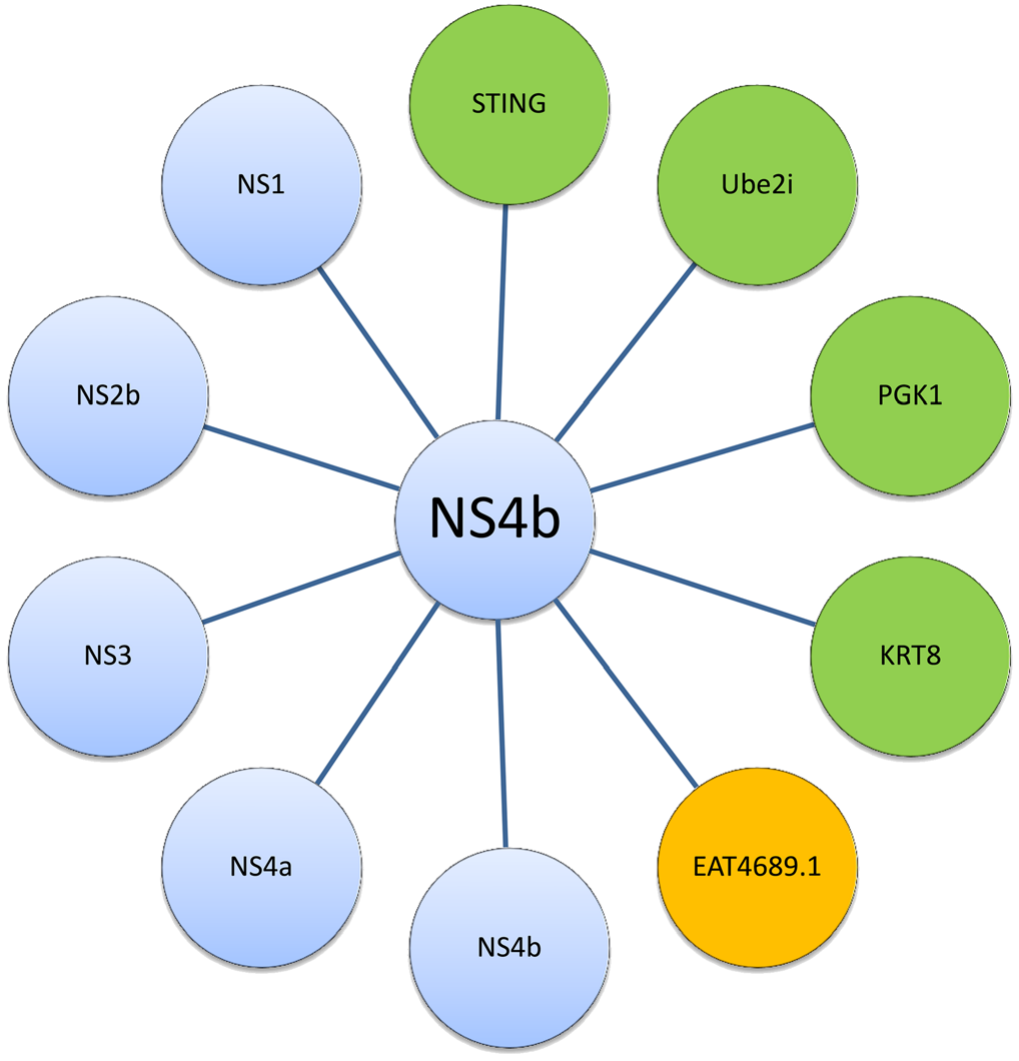
Interactomics of flaviviral NS4b. NS4b interacts with several viral NS proteins (blue), as well as host cell proteins of human (green) and mosquito cells (orange). KRT8, type II cytoskeletal 8 keratin; EAT45689.1, phosphoglycerate transporter EAT45689.1; PGK1, phosphoglycerate kinase 1; STING, stimulator of interferon genes protein

Non‐structural 4b proteins encoded by different flaviviruses share the same predicted topology, with five integral transmembrane segments 30 (Figures 2A,B and 3). In DENV‐2‐infected cells, the NS4b protein localizes to the perinuclear region of the cell 30 with the transient appearance of dot‐like cytoplasmic foci early during the course of infection, which corresponds to several distinct fairly complex membrane structures: one‐membrane and two‐membrane vesicles, convoluted membranes, and finally virions that are closely associated with cisternae reminiscent of the rough ER 33. NS4b does not co‐localize with lipid droplets (cytoplasmic lipid droplets play a role in the production of infectious DENV and HCV particles) or Golgi markers. The protein rather accumulates at, or close to, the ER and ER‐derived membranes and together with NS proteins and the DENV double‐stranded RNA (dsRNA), suggesting that NS4b takes part in the replication of the virus, rather than in a later stage of viral assembly 30, 34. In WNV‐infected cells, a fraction of NS4b was originally described to localize to the nuclear region as well 35, but the functional relevance of this observation was never explored. Genetic and biochemical evidence suggests that NS4b binds to, and interacts with, the NS1 protein with the NS4b domains involved oriented to the luminal side of the ER (Figures 2A and 4 and Table 1) 36. NS4b and NS2b of WNV were shown to interact using fluorescence resonance energy transfer technology (Figure 2A and Table 1) 37. The DENV‐2 NS4b interacts with the cytoplasmic NS3, and data from dsRNA unwinding assays suggest that NS4b may assist the NS3 helicase to dissociate from single‐stranded RNA 34 (Figures 2A and 4 and Table 1). Subdomains 2 and 3 of the NS3 helicase region and the 30aa cytoplasmic loop of NS4b are required for binding 38. Interaction between membrane proteins NS4a and NS4b of DENV‐2 39 and JEV 40 was reported (Figure 2A and Table 1). Dimerization of DENV‐2 NS4b has recently been reported, with the cytosolic loop (amino acids 129–165) and the C‐terminal region (amino acids 166–248) being essential for oligomerization 41. These data are in line with biophysical assays showing a sufficient proximity and an interaction between differentially tagged NS4b variants of WNV 37 (Figures 2A and 4 and Table 1).

**Table 1:** Interactomics of flaviviral NS4b

Protein	Origin/host	Protein function	Methods of interaction characterization	Residues involved in the interaction	Residues of NS4b involved in the interaction	Function of the interaction for the viral life cycle	Study
NS1	Flavivirus	Component of the membrane‐bound viral RC, immune pathogenesis	Genetic link; co‐immunoprecipitation (co‐IP)	RQ10NK	F86C	Possibly needed for formation of functional RCs	35
NS2b	Flavivirus	Component of the membrane‐bound viral RC; virus assembly and/or release	Biomolecular fluorescence complementation; fluorescence resonance energy transfer (FRET)	Unknown	Unknown	Unknown	37
NS3	Flavivirus	Protease, helicase/nucleoside triphosphatase, RNA 5′‐triphosphatase	Yeast two‐hybrid; co‐localization; co‐IP; *in situ* proximity ligation assay; surface plasmon resonance; nuclear magnetic resonance (NMR); functional studies (helicase unwinding assay)	C‐terminal part of NS3 (amino acids 303–618) that contains a helicase motif; subdomains 2 and 3 of the NS3 helicase 181 region	Cytoplasmic loop [134]; P140L abolishes interaction; possibly interaction is conformation dependent [35]	Possibly NS4b enhances NS3 helicase activity to unwind dsRNA to dissociate from single‐stranded RNA	34, 38
NS4a	Flavivirus	Virus‐induced membrane alterations	Yeast two‐hybrid; co‐IP; NMR; genetic link	Amino acids 40–76 (spanning the first transmembrane domain [amino acids 50–73])	Amino acids 84–146 (also spanning the first transmembrane domain [amino acids 101–129])	Unknown	39, 40
NS4b	Flavivirus	IFN‐α/β antagonist, RNAi suppressor, stress granules and UPR modulator, viral adaptability	FRET; protein dimerization (gel filtration, chemical cross‐linking, and multi‐angle light scattering); genetic link	Cytoplasmic loop and C‐terminal region		Possibly, dimer is needed for proper protein folding and functioning	37, 41
STING	Human	Stimulator of the IFN genes proteins	Bioinformatics analysis; co‐IP; co‐localization	Amino acids 125–222	Amino acids 1–97 (YFV) and 1–94 (DENV)	YFV NS4b blocks RIG‐I‐mediated IFN signaling by binding to STING	42
Ube2i	Human	SUMO‐conjugating enzyme Ube2i, which catalyzes the transfer of SUMO to its target proteins	Yeast two‐hybrid; small interfering RNA knockdown	Unknown	Cytoplasmic loop	Unknown	43, 44
PGK1	Human	Phosphoglycerate kinase, a glycolytic enzyme that catalyzes the conversion of 1,3‐diphosphoglycerate to 3‐phosphoglycerate	Yeast two‐hybrid	Unknown	N‐terminal part of protein	Unknown	43
KRT8	Human	Member of the type II keratin family	Yeast two‐hybrid	Unknown	C‐terminal part of protein	Unknown	43
EAT4689.1	Mosquito cells	Member of the phosphoglycerate transporter family	Yeast two‐hybrid	Unknown	Unknown	Unknown	45

In a first effort using complementary replication‐deficient WNV replicons, NS4b (along with NS2a, NS2b, and NS4a) could not be complemented in *trans*, in contrast to the soluble NS1, NS3, and NS5 proteins 46. A more recent report suggests that DENV‐2 NS4b harboring a P104R mutation that renders the protein nonfunctional for intracellular replication can be (at least to some extent) complemented in *trans* 29. In another report, the NS4b lethal K143A mutation could only be rescued by the DENV‐2 replicon that expressed the wild‐type NS4b, but not by a heterologous expression. This suggests that *trans*‐complementation of (at least some) lethal NS4b mutants requires the context of an intact RC 41. This would be perfectly in line with 2K‐NS4b being co‐translationally processed and assembled in close proximity to other components of the viral replication machinery, thereby providing a chaperoning framework for full functional maturation. Moreover, NS4b may readily be considered to consist of several separate functional domains (Figure 2A), each of which may act independently from the rest of the protein (and is hence more likely to be *trans*‐complemented than others).

The NS4b protein is highly conserved (Figure 3); among the DENV serotypes, an overall protein sequence conservation of 78% is calculated (within a serotype, this is 97%) 47. On the other hand, the flaviviral NS4b seems to serve as a mutational hotspot and shows a high likeliness and frequency of acquiring adaptive mutations in response to varying selective pressures (Table 2 and Figure 2A). DENV‐2 replicons passaged in Vero cells acquired the NS4b T108M mutation, a change that enhances viral RNA replication in a cell type‐dependent manner 48. The P101L mutation in DENV‐4 NS4b results in a small plaque phenotype in the C6/36 mosquito cell line, whereas it increases the plaque size in Vero and HuH‐7 cells 49. The NS4b L112F mutation was observed in DENV‐4 passaged in Vero cells 50. The NS4b V109A, G119S, and L112F mutations increase (independently) the replication kinetics of DENV‐4 in Vero cells 50. Chimeric virus expressing WNV structural proteins in a DENV‐4 backbone carries two mutations in the NS4b protein (T105I and L112S) that were shown to be responsible for reduced peripheral virulence and neurovirulence 51. The K143A mutation in the cytoplasmic loop of DENV‐2 NS4b completely abrogates viral replication in cell culture 41. The DENV‐2 isolate D2Y98P with the mutation in NS4b results in robust replication and mortality in AG129 (IFN‐α/β and IFN‐γ receptor knockout) mice despite the fact that the virus is not mouse adapted. Its lethal phenotype can be abolished, and its high pathogenicity reverted by a single F52L mutation in the NS4b protein sequence. Conversely, the reverse L52F mutation made the DENV‐2 strain TSV01 (which does not replicate well in mice) replicate efficiently and cause mortality in mice 52. Moreover, the NS4b V115A in DENV‐2 strain D2S10 (together with the K122I mutation in the envelope protein) causes mortality at 10‐fold lower inoculum in mice than in wild‐type virus 53. Similar to DENV, adapting the YFV 17D to Vero cells resulted in acquisition of the I113M mutation in the NS4b 54. The NS4b V98I mutation was associated with more aggressive pathogenesis and lethal viscerotropic disease in the YFV Asibi hamster model 55. Passaging the yellow fever wild‐type strain Asibi in HeLa cells attenuated the virus for monkeys and newborn mice and resulted in loss of infectivity for mosquito vectors. Three out of 10 acquired mutations were mapped to the NS4b protein (I95M, V98I, and E144K) 56. The L204S NS4b mutation is present in the highly neuroattenuated YFV 17D vaccine 57. The Q136K mutation in NS4b protein appears in YFV 17D chimera with prM and protein E from the Modoc virus 58. The NS4b mutation P38G in WNV (isolate NY99) results in attenuation of neuroinvasiveness in mice 57. For some WNV NS4b mutations (such as C102S or E249G), a direct link can be established between slower viral growth kinetics and lower viral yields in cell culture and virulence phenotypes in mice 59, 60. Of note, the WNV NS4b E249G mutation is a mouse‐adapted mutation that attenuates *in vitro* virus synthesis. Interestingly, a second site mutation in close proximity (L246M/N/Q) can fully restore viral replication in a cell culture, further stressing the important role of NS4b for flaviviral adaptation 61. The F86C mutation in NS4b is a suppressor mutation that rescues the replication of the NS1 RQ10NK mutant 36. The live‐attenuated JEV vaccine strain SA14‐14‐2 carries an I106A substitution in NS4b 62. Overall, seemingly small changes in the primary sequence of the flaviviral NS4b protein may cause marked changes in replication capacity and pathogenic potential of the virus, pointing to the key role of this protein in flaviviral replication.

**Table 2:** Functional relevance of NS4b mutations for flaviviral replication

Mutation	Virus	Serotype	Phenotype	Study
P38G	WNV	NY99	Temperature‐sensitive replication and smaller plaques *in vitro*; lowers viremia and attenuated neuroinvasiveness in mice; mice infected with NS4b P38G mutant show stronger effector and memory T responses	60
L52F	DENV	Serotype 2	Non‐mice‐adapted clinical isolate; increased viral titer and mortality in mice; L52F mutation in the NS4B of the non‐virulent DENV‐2 strain TSV01 led to 80% lethality and increased viremia in IFN knockout mice	52
F86C	WNV	NY99	Suppressor mutation that rescues replication of NS1 mutant (RQ10NK)	36
I95M	YFV17D	French neurotropic vaccine strain; Asibi HeLa‐adapted Asibi strain p6	Adaptive mutation that arises in the HeLa cell culture, but it is not present in infected mosquitos	56
V98I	YFV	Asibi	Mutation generated by *in vivo* passaging virus 7 (hamsters) hamsters; it increases the pathogenesis in Syrian golden hamsters	55
P101L	DENV	DENV‐4 serotype 4	Decreased replication in C6/36 cells and in decreased infectivity for mosquitoes; enhanced replication in Vero (African green monkey) and HuH‐7 (human hepatoma cells) and enhanced replication in severe combined immunodeficient (SCID) mice implanted with HuH‐7 cells (SCID‐HuH‐7 mice)	49
C102S	WNV	WNV infectious clone WN/IC P991	Temperature sensitive at 41 °C *in vitro*; attenuation of the neuroinvasiveness and neurovirulence phenotypes in mice	59
T105I	WNV–DENV‐4 chimera	DENV‐4	Adaptive mutation arising in DENV‐4 carrying the prM/E of WNV; shown to be responsible for reduced peripheral virulence and neurovirulence	51
I106A	JEV	JEV strain SA14	Mutation that provides a molecular basis of attenuation of neurovirulence of wild‐type JEV strain SA14	62
T108M	DENV	A stable luciferase reporter DENV‐2	Mutation enhances viral RNA replication in a cell type‐specific manner	48
V109	DENV	DENV‐4	Adaptive mutation that enhances virus replication in Vero cells	50
L112F	DENV‐4	DENV‐4 strain 814669 (Dominica, 1981)	Adaptive mutation that enhances virus replication in Vero cells	50
L112S	WNV–DENV‐4 chimera	DENV‐4	Adaptive mutation arising in DENV‐4 carrying the prM/E of WNV; shown to be responsible for reduced peripheral virulence and neurovirulence	51
I113M	YFV	17D	Mutation seems to be required for virus adaptation to Vero cells	54
V115A	DENV	DENV‐2 strain (D220), generated from D2S10 strain (GenBank: JF730054.1)	Adaptive mutation, acquired via serial passaging that (together with K122I in the envelope protein) appears to account for the observed induced mortality at 10‐fold lower doses than D2S10 in mice lacking only the IFN‐α/β receptor in C57BL/6 or 129 backgrounds under both non‐enhanced and antibody‐enhanced conditions	53
G119S	DENV	DENV‐4	Adaptive mutation that enhances virus replication in Vero cells	51
Q136K	YFV–Modoc virus chimera	YFV17D	Adaptive mutation that results in a YFV17D with pM and the E protein of Modoc virus; chimera virus in neuroinvasive SCID mice	58
K143A	DENV	Serotype 2	Lethal mutation discovered by alanine scanning of the cytoplasmic loop	41
E144K	YFV	Asibi	Mutation involved in the attenuation of the virulence of wild‐type strain Asibi	56
L204S	YFV	YFV‐17D (RK 168‐73 vaccine reference strain from Robert Koch Institute)	Mutation appears to lower neurovirulence (together with mutations in envelope protein N153S)	57
E249G	WNV	YFV‐17D vaccine strain	Mouse‐adapted mutation that attenuates virus synthesis; smaller plaques, slower growth kinetics, and lower RNA synthesis *in vitro* in a host‐dependent manner, with the greatest difference in rodent cells (C3H/He and BHK‐21) and the least difference in mosquito cells (C3/36); second site mutation at residue 246, compensates for the low‐replication phenotype in cell culture	61

## Interaction with Host Proteins

An increasing number of studies report on host proteins that interact with flaviviral proteins 43, 44, 63, 64. By using a genome‐wide yeast two‐hybrid screen, interacting partners of DENV‐2 NS4b were identified: phosphoglycerate kinase (PGK1), type II cytoskeletal 8 keratin (KRT8), and the small ubiquitin‐like modifier (SUMO)‐conjugating enzyme Ube2i (originally called ubiquitin‐conjugating enzyme E2I) (Figures 2A and 4 and Table 1). Functional data on the role of PGK1 and KRT8 on the flaviviral life cycle are missing. Intriguingly, enzymes from the glycolytic pathway (such as PGK1 and glyceraldehyde 3‐phosphate dehydrogenase) are critical for the replication of RNA viruses 65. RNA viruses may thus rely on highly conserved metabolic pathways as scaffolds to accommodate their own intracellular processes. Accordingly, a member of the phosphoglycerate transporter family (EAT45689.1) from the DENV and YFV vector *Aedes aegypti* was shown to interact with NS4b, but the functional relevance of this interaction remains unknown 45.

Ube2i, which catalyzes the transfer of SUMO to its target proteins 66, was shown to constitute an essential host factor needed for DENV replication 63. Ube2i also interacted with the DENV‐2 envelope protein 67, and the overexpression of Ube2i reduced plaque count of DENV‐2 in mammalian cells. SUMOylation has been shown for a number of viruses to play an important role at the host–pathogen interface 68, 69. The replication of DNA viruses such as human cytomegalovirus 70, HSV 71, and the chicken embryo lethal orphan adenovirus 72 is affected by SUMOylation. As to what concerns RNA viruses, the Ebola virus VP35 protein was shown to bind to the IFN regulatory factor 7 (IRF7) and to recruit Ubc9 and the SUMO E3 ligase PIAS‐1 (originally called *p*rotein *i*nhibitor of *a*ctivated STAT‐1 [signal transducer and activator of transcription 1]) that enhances the activity of Ube2i, which in turn SUMOylates IRF7. By doing so, type I IFN production is blocked, allowing viral evasion of innate immunity 73. The 3C protease of enterovirus 71 (EV71) is a direct substrate of Ube2i SUMOylation, and 3C SUMOylation decreases its cellular half‐life 74. In contrast, impaired SUMOylation of EV71 3C was shown to stimulate viral replication and apoptosis of the host cell, and SUMOylation‐deficient EV71 clinical isolates were shown to invade the CNS 74. Similarly, the interaction between NS4b and Ube2i may be crucial during DENV (and flavivirus) infection and pathogenesis. Of note, SUMOylation plays an important role in epigenetic control of cellular transcription and oncogenesis and is therefore judged to be a promising target for cancer therapy 75, 76. In turn, anticancer drugs interfering with cellular SUMOylation might deserve consideration of a second medical use as future inhibitors of flaviviral replication.

The NS4b protein shares homology with several viral and cellular proteins. The NS4b of the WNV (Kunjin strain) shares homology with nuclear export signals of the HIV shuttle protein Rev and the protein kinase inhibitor 35, 77. The flavivirus NS4b exhibits homology to *s*timulator of *t*he IFN *g*enes proteins (STING) 42, in the region that is critical for STING function. Moreover, YFV NS4b was reported to block retinoic acid‐inducible gene I (RIG‐I)‐mediated IFN signaling by binding to STING 78. The results obtained for YFV could not be replicated for DENV 76. In contrast, the DENV‐2 NS2b/NS3 protease was shown to inhibit IFN production by cleaving human STING, and STING knockdown following DENV‐2 infection reduces IRF3 activation and IFN induction 78.

## Immune Evasion Mechanisms

### Evidence for the inhibition of IFN signaling by NS4b

The IFN response is an initial and essential host defense mechanism against many viruses, including flaviviruses 79, 80. Mammalian cells recognize viral RNAs by specific pattern recognition receptors (PRRs), primarily by the endosomal Toll‐like receptors 3 and 7 and the cytoplasmic RNA sensors RIG‐I and melanoma differentiation‐associated protein 5 (MDA‐5). Binding of the viral RNAs to these PRRs results in activation of transcription factors, such as IRF3 and IRF7 and nuclear factor kappa‐light‐chain‐enhancer of activated B cells NF‐kB, and finally the induction of IFN‐α and IFN‐β. Secretion of IFNs is followed by engagement of the IFN‐α/β receptor in an autocrine and paracrine fashion that activates Janus kinase‐signal transducer and activator of transcription (JAK‐STAT)‐dependent and independent signal transduction cascades. This induces the expression of hundreds of IFN‐stimulated genes (ISGs), a subset of which has antiviral properties against flaviviruses 80, 81. In order to prevent the induction of these antiviral ISGs, flaviviruses target JAK‐STAT signaling pathways, and several NS proteins of flaviviruses serve as specific IFN antagonists. Initial heterologous expression of NS2a, NS4a, or NS4b enhanced replication of an IFN‐sensitive sentinel virus by blocking nuclear localization of STAT‐1 82. Subsequent experiments revealed that NS4b of DENV, WNV, and YFV partially blocks STAT‐1 activation and thus ISG induction. Expression of the precursor NS4a/b fusion protein does not cause an inhibition of IFN signaling unless this product is cleaved by the viral NS2b/3 protease, indicating that proper viral polyprotein processing is required for the anti‐IFN activity of NS4b. The first 125 amino acids of DENV‐2 NS4b are sufficient for inhibition of IFN‐α/β signaling, with residues located between amino acids 77 and 125 that are most likely oriented to the ER luminal side 30, 41 playing an essential role 82. Likewise, E22 and K24 residues in the NS4b of WNV were shown to control IFN resistance in cells expressing subgenomic replicons 83. This was however not confirmed in cells expressing infectious virus, suggesting an independent role of (other) NS or structural genes in the IFN inhibition at different steps of the flaviviral life cycle 83. Determining the inhibition of IFN signaling by different flaviviruses is further complicated by strain‐specific (but not serotype‐specific) differences regarding their suppression of IFN signaling. For instance, some DENV strains belonging to different serotypes (TSV01 [DENV‐2], SG167 [DENV‐1], MY02569 [DENV‐1], MY10340 [DENV‐2]) were shown not to inhibit STAT‐1 upon stimulation with IFN‐β, whereas others (NGC [DENV‐2], MY10245 [DENV‐1], MY22563 [DENV‐2], and MY22713 [DENV‐4]) readily exhibit such a suppressive activity 84. Minor sequence variation mapping to the N‐terminal half of NS4b can only partly explain these obvious differences. Thus, the full picture of IFN antagonist function of NS4b is still missing. The role that different viral (such as NS2b/3, 4a, and 5) 78, 81, 85, 86, 87 and host proteins (e.g., regulators of STAT‐1, such as Ube2i and PIAS‐1) 88, 89, 90 play in this process remains to be explored. Similar to the large impact, subtle changes to either component (such as single‐point mutations in NS4b, see earlier discussion) may have an impact on the overall function of the flaviviral RC, thereby resulting in largely altered viral growth kinetics and pathogenesis. It might be assumed that the subversion of IFN signaling by flaviviruses is orchestrated by an equally well‐balanced interplay of multiple viral and host factors, with NS4b possibly again acting as a central player.

### Evidence for suppression of RNAi by NS4b

RNA interference (RNAi) is an evolutionary conserved mechanism that targets endogenously expressed RNAs in a sequence‐specific way and plays an important role in the host cell defense against viral pathogens (and transposons) in plants, insects nematodes, and mammals 91. In short, during viral infection, long dsRNA is recognized as a specific pathogen‐associated molecular pattern and chopped into a pool of 21‐nucleotide small interfering RNAs (siRNAs) by the resilient dsRNA‐specific RNase Dicer. These siRNAs then induce and target the RNA‐induced silencing complex to their cognate mRNA for endoribonucleic cleavage or translational arrest. Flaviviruses evolved mechanisms in which they are able to suppress elements of the RNAi machinery (reviewed in 92). For instance DENV infection of HuH‐7 hepatoma cells leads to a downregulation of several key components of the host RNAi machinery, such as Dicer, Drosha, and Argonaute proteins 1 and 2; conversely, siRNA‐mediated silencing of these host factors leads to an increase in DENV replication 93. Suppression of RNAi could be mechanistically explained by viral RNA decoys that are expressed by all flaviviruses 93. However, NS4b from all four serotypes of DENV was recently demonstrated to act as a potent viral suppressor of RNA silencing (VSRs) 94 similar to those found in some plant 95 and insect viruses 96. The molecular mechanism by which NS4b inhibits RNAi seems to differ from other VSRs as it does not bind dsRNA/siRNA but rather inhibits Dicer activity and thus siRNA generation 94. Mutagenesis studies revealed that this RNAi suppressor activity depends on the predicted transmembrane regions 3 and 5 of NS4b (Figure 2A, TMD3 and TMD5).

### NS4b as modulator of SG and UPR

Stress granules (SG) are dense yet dynamic cytoplasmic aggregations (100–200 nm) often associated with the ER, composed of proteins and RNAs that appear when cells are exposed to certain stress 97, 98. During SG formation, translation of many proteins is inhibited by phosphorylation of eukaryotic elongation factor 2α, which in turn leads to ablation of elF2‐GTP‐tRNA dependent translation initiation and polysome disassembly 98. In an arms race, many viruses, such as poliovirus and cardiovirus (*Picornaviridae*), Chikungunya virus (alphavirus), Junin virus (*Arenaviridae*), orthoreovirus and rotavirus (dsRNA viruses), and HIV‐1 (retrovirus), evolved means to block this response (reviewed in 99), indirectly corroborating the importance of SG for antiviral immunity. WNV NS4b (together with other NS proteins) was shown to suppress early viral RNA synthesis and membrane remodeling during natural WNV infections and consequently evade and even suppress experimentally induced SG formation 100.

NS4b may play a similar role in the unfolded protein response (UPR) that is a coordinated change at another level of gene expression triggered by perturbations in functions of the ER 101, 102. Mechanistically, three different arms of UPR can be activated: (i) IRE‐1‐induced splicing event of the X‐box binding protein 1 (Xbp‐1) mRNA that results in its activation as a transcription factor, which in turn leads to the expression of chaperones and host factors (such as DnaJ/Hsp40‐like genes, p58IPK, ERdj4, HEDJ, EDEM, and protein disulfide isomerase P5) 103, 104 involved in quality control of cellular protein synthesis 104; (ii) the protein kinase R‐like ER kinase (PERK); and (iii) activating transcription factor 6 (ATF6). The WNV NS4b (together with NS2b and NS4a) was shown to activate the ATF6/IRE‐1 pathways, resulting in Xbp‐1 transcription and splicing; this may aid in the proliferation of ER membranes for the RC 103, 105. The effects of DENV on the UPR are time dependent and possibly cell type specific 106. Of note, inhibition of protein folding and post‐translational quality control by selective blocking of ER resident cellular α‐glucosidases (by castanospermine and other related compounds) were shown to exert potent *in vitro* antiviral activity against several flaviviruses including DENV (four serotypes), JEV, and WNV 107, 108, 109, 110, 111, 112. It was recently shown that *N*‐(4‐hydroxyphenyl)retinamide activates PERK, thereby clearing the virus in DENV‐2‐infected cells at non‐toxic/apoptotic concentrations. The compound also resulted in some level of protection in DENV‐induced morbidity and mortality in mice 113. This stresses the importance of interfering with post‐translational regulation of protein expression, in particular of conquering the ER compartments by the activity of the NS4b and other viral factors to aid flaviviral replication.

### Small‐molecule inhibitors of flavivirus replication that target NS4b

Several phenotypic cell‐based screens for inhibitors of flaviviral replication resulted in the identification of molecules that target NS4b (Figure 2B and Table 3). The plant alkaloid lycorine inhibited the replication of WNV, YFV 31, and DENV 114. For WNV, resistance to lycorine was conferred by a V9M mutation in the viral 2K peptide 31. The exact molecular mechanism of this antiviral activity has not yet been solved, yet it is tempting to speculate that lycorine interferes with co‐translational membrane insertion and signalase processing of the 2K/NS4b protein, thus targeting a host process rather than acting directly on the viral target (especially considering its poor selectivity and pronounced cellular toxicity) 31. Using subgenomic replicons in the high‐throughput screen, two potent small‐molecule inhibitors of YFV replication were identified (CCG‐4088 and CCG‐3394) 115, which select for a single lysine‐to‐arginine mutation (K128R), conferring compound resistance that maps to the NS4b region. This position according to the proposed topology 30, 41 is located in the transmembrane domain 3 of the NS4b protein (Figure 2B), embedded in the ER membrane. Another small‐molecule inhibitor, Novartis Institute for Tropical Diseases (NITD)‐618, inhibits four serotypes of DENV at low micromolar concentrations 29. NITD‐618 resistance is conferred by mutations P104L and A119T that map to the TMD3 (Figure 2B). Interestingly, the mutated 104L residue exists as the wild type in other flaviviruses such as in the JEV and WNV NS4b proteins, possibly accounting for the selectivity of NITD‐618 in inhibiting only DENV. A δ opioid receptor antagonist (SDM25N) was found to restrict genomic DENV‐2 RNA replication in a cell type‐specific manner, and the F164L mutation at the end of the cytoplasmic domain located between TMD3 and TMD4 of NS4b protein (Figure 2B) conferred resistance to SDM25N 116. Remarkably, an NS4b amino acid substitution at P104, which was previously shown to confer resistance to the DENV inhibitor NITD‐618 29 also provided cross‐resistance to SDM25N 116, arguing for a shared molecular mechanism of action. Likewise, both the TMD3 and the cytoplasmic loop have been implemented in dimerization of NS4b 41, and dimerization of other viral transmembrane proteins such as the (notably non‐homologous) NS4b protein of the HCV (discussed later) has, at least conceptionally, been proven to serve as a viable molecular target for antiviral inhibition 117. Finally, dasatinib, an oral multi‐BCR/Abl and Src family tyrosine kinase inhibitor (approved for first‐line use in patients with chronic myelogenous leukemia), was shown to inhibit *in vitro* DENV replication, and a drug‐resistant variant that confers resistance was shown to carry a mutation in NS4b (T108I) 118. This mutation intriguingly maps again to pTMD3. Following large CPE‐based screening effort (employing a highly diverse small compound library) and extensive hit‐to‐lead optimization, our laboratory identified a novel class of highly potent (low nanomolar) inhibitors of *in vitro* DENV replication that targets NS4b (our unpublished data). The compounds exert pan‐serotype activity resulting in >4log_10_ reduction of viral RNA yield; in most cases, the reduction is even to undetectable levels (as determined by RT‐qPCR). Compounds belonging to this class efficiently reduced viral replication in DENV‐infected mice. For this class of compounds, cross‐resistance is not observed with other NS4b‐targeting compounds such as lycorine 31 and NITD‐618 29, arguing for a unique molecular mechanism of action.

**Table 3:** Anti‐flaviviral compounds that target NS4b

Virus (serotype/strain)	Compound	Structure	Mechanism of action	Publication
DENV‐2, WNV, YFV	Lycorine	(1) 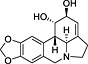	Resistance mutation V9M in 2K peptide of WNV	31
YFV	CCG‐4088	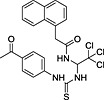	Resistance mutation K128R	115
YFV	CCG‐3394	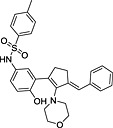	Resistance mutation K128R	115
DENV‐2 replicon	NITD‐618	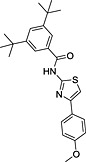	Resistance mutations 104L and A119T	29
DENV‐2 replicon	SDM25N	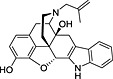	Resistance mutation F164L cross‐resistant with NITD‐618	116
DENV‐2	Dasatinib	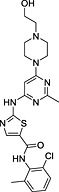	Inhibition by AZD0530, dasatinib, and RNAi‐mediated Fyn kinase knockdown can be counteracted by mutation T108L	118
DENV‐2	AZD0530	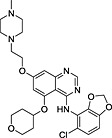		

### Flavivirus NS4b and HCV NS4b—same name, different proteins

**Figure 5:**
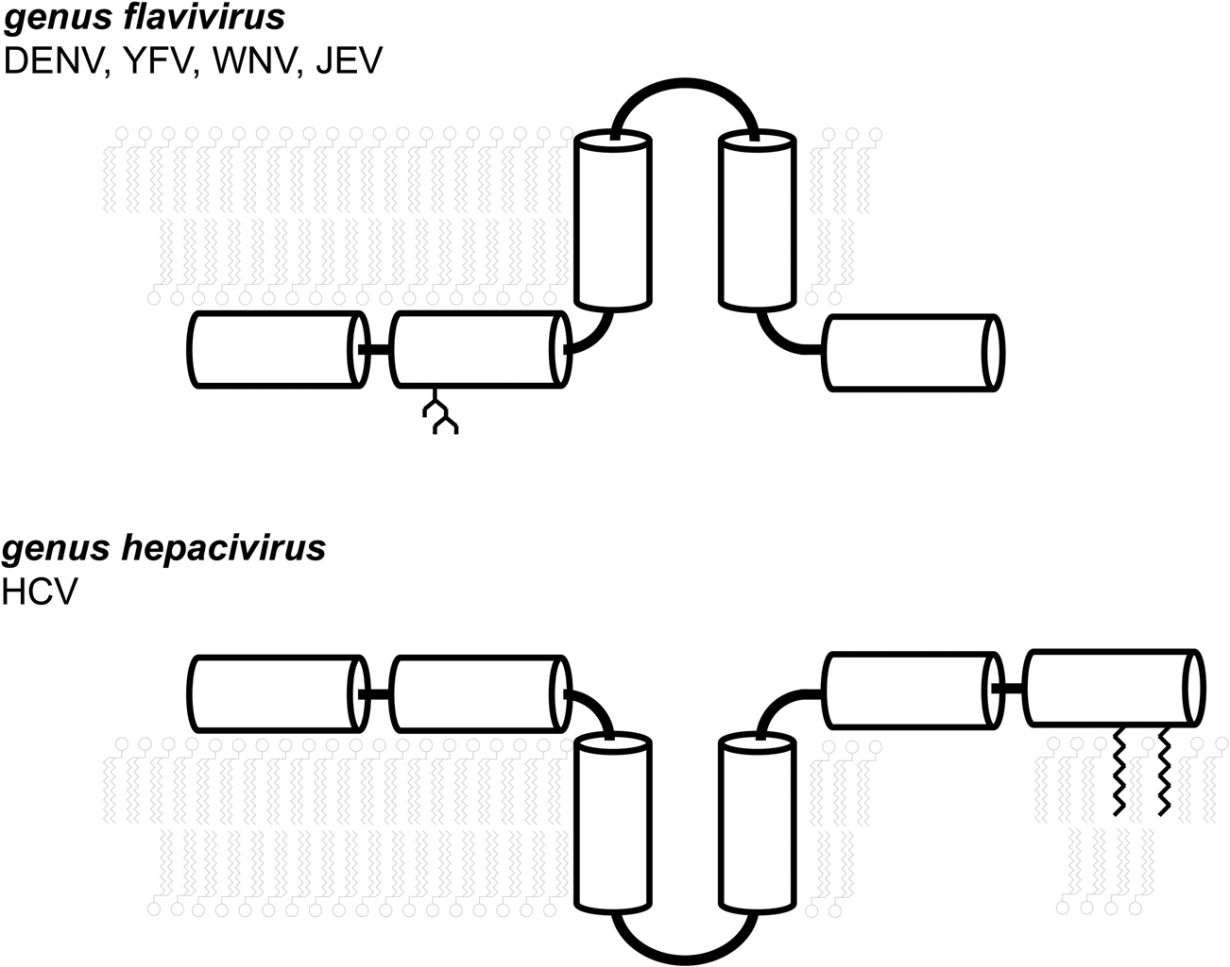
Structural differences between the NS4b proteins of *Flaviviridae*. The NS4b of HCV (lower panel) belonging to another genus *Hepacivirus* with the common *Flaviviridae* family differs from the NS4b of the *Flavivirus* genus (e.g., DENV, YFV, WNV, and JEV). HCV NS4b consists of six α‐helices, of which the membrane topology can be considered a mirror image of the flavivirus NS4b with both N and C termini exposed to the cytoplasmic leaf of the ER membrane. HCV is palmitoylated, and homodimers stabilized by intermolecular disulfide bonds cross‐linking the N‐terminal helices

The NS4b proteins of flaviviruses such as DENV or WNV and of the HCV (another member of the *Flaviviridae* family) are orthologous (genes encoded at the same position of the viral ORF) but not homologous (evolutionarily related), either in their structure or in the function they play in the respective viral life cycles (Figure 5). HCV NS4b is an integral membrane protein containing three structural domains 119. The protein is necessary and sufficient to induce the intracellular membrane alterations known as a membraneous web that harbors the viral RCs 120, 121. The N‐terminal part of HCV NS4b contains two amphipathic α‐helices of which the second was found to have the potential to traverse the phospholipid bilayer as a transmembrane segment, most probably upon oligomerization 122. The central part of HCV NS4b is predicted to comprise four transmembrane segments. The C‐terminal part consists of a predicted highly conserved α‐helix, a membrane‐associated amphipathic α‐helix, and two reported palmitoylation sites, but the function of these modifications remains to be explored further 119, 123. HCV NS4b interacts with other viral NS proteins (NS3‐4a, NS5a, and NS5b) and has been reported to bind viral RNA 124.Besides, HCV NS4b was found to harbor a nucleoside triphosphatase activity 120, 124 and to have a role in viral assembly 121. Inhibition of STAT‐1/IFN signaling appears to not be a major function of the HCV NS4b 125. However, similar to the flavivirus NS4b 101, HCV NS4b is a strong regulator of UPR signaling 126. Inhibitors of HCV replication may interfere with NS4b dimerization 117. Finally, the HCV NS4b inhibitor clemizole (clemizole hydrochloride) 127 was positively evaluated in the phase I clinical trial with treatment‐naïve HCV patients.

## Concluding Remarks

The majority of Food and Drug Administration‐approved antiviral drugs target enzymatic functions of viral proteins, and this includes the following: (i) nucleoside analogue inhibition of the HSV DNA polymerase, nucleoside, nucleotide analogues, and non‐nucleoside inhibition of the HIV and HBV and of the HCV RdRp; (ii) inhibition of the HIV protease and HIV integrase inhibitors. Other antiviral targets are structural proteins that are involved in viral entry, fusion, or maturation. The flaviviral NS4b, to which also no enzymatic activity has been described, does not belong to any of these categories. In this respect, the flavivirus NS4b can be compared with the HCV NS5a protein. This HCV protein with a pivotal role in HCV replication has been shown to be an excellent target for inhibition viral replication 128. The HCV NS5a is a 447‐amino‐acid, zinc‐binding phosphoprotein that, with no enzymatic function, interacts with several viral and host proteins (including cyclophilin A and Raf‐1 kinase), and it is a key player in viral replication. It binds viral RNA directly and also inhibits type I IFN signaling. A number of drugs (some of which have picomolar potency) are or have been developed for the treatment of HCV infection, including but not limited to daclatasvir (BMS‐790052), ledipasvir (GS‐5885), and ABT‐267 129.

Likewise, NS4b associates with the flaviviral RCs and acts as a modulator of innate immune responses. Furthermore, mutations in NS4b appear during adaptation to the growth in different hosts and changes viral replication kinetics and pathogenesis. The multiple and often “surprising” contributions of NS4b make it a true “chameleon” and “jack in the box.” Given, akin to NS5a, its key role in viral replication, it is not surprising that NS4b appears as an excellent target for inhibition of viral replication. It seems likely that more NS4b inhibitors may be developed in the future for the treatment and/or prophylaxis of flavivirus replication. Such inhibitors may also serve as chemical probes to help understand the precise role of NS4b in the complex molecular biology, immunology, and immunological responses (or their deficiencies) during flaviviral replication.

## Conflict of Interest

The authors have no competing interest.
